# Stark Effect Control of the Scattering Properties of Plasmonic Nanogaps Containing an Organic Semiconductor

**DOI:** 10.1021/acsaom.2c00135

**Published:** 2022-12-20

**Authors:** Donatello Pagnotto, Alina Muravitskaya, David M. Benoit, Jean-Sebastien G. Bouillard, Ali M. Adawi

**Affiliations:** † Department of Physics and Mathematics, University of Hull, Cottingham Road, HullHU6 7RX, United Kingdom; ‡ G. W. Gray Centre for Advanced Materials, University of Hull, Cottingham Road, HullHU6 7RX, United Kingdom

**Keywords:** Stark effect, plasmonic nanogap, organic semiconductor, FDTD, molecular polarizability, DFPT

## Abstract

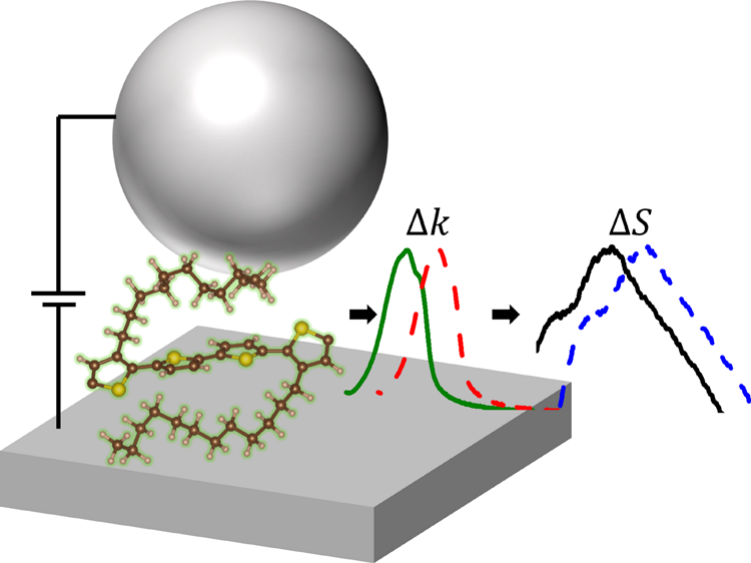

The development of actively tunable plasmonic nanostructures enables real-time reconfigurable and on-demand enhancement of optical signals. This is an essential requirement for a wide range of applications such as sensing and nanophotonic devices, for which electrically driven tunability is required. By modifying the transition energies of a material via the application of an electric field, the Stark effect offers a reliable and practical approach to achieve such tunability. In this work, we report on the use of the Stark effect to control the scattering response of a plasmonic nanogap formed between a silver nanoparticle and an extended silver film separated by a thin layer of the organic semiconductor PQT-12. The plasmonic response of such nanoscattering sources follows the quadratic Stark shift. In addition, our approach allows one to experimentally determine the polarizability of the semiconductor material embedded in the nanogap region, offering a new approach to probe the excitonic properties of extremely thin semiconducting materials such as 2D materials under applied external electric field with nanoscale resolution.

## 1 Introduction

Plasmonic nanogaps offer unique optical properties such as a wide range of tunable plasmonic modes with different polarization, near-field and far-field optical characteristics,^1−6^ confined electromagnetic field at nanometer length scales,^7−9^ greatly enhanced electromagnetic fields,^2^ and increased local density of optical states.^6,10−14^ These properties have boosted their use in a wide range of applications such as chemical/biological sensors,^15,16^ imaging,^17^ ultracompact optoelectronic devices,^18−21^ active nanopixels,^22^ optical elements,^23^ and information technology.^24^ While the near-field and far-field optical response of plasmonic nanogaps can be tuned by carefully controlling their geometrical parameters,^2,4−6,9^ it is difficult to change them in real time.^25^ However, the ability to dynamically modulate their optical responses is highly desirable,^26^ as the development of actively tunable plasmonic nanostructures enables real-time reconfigurable and on-demand enhancement of optical signals, a prerequisite for a wide range of applications such as plasmonic sensing,^27,28^ and nanophotonic devices.^22,29,30^

A variety of modulation methods have been explored to develop dynamically tunable plasmonic nanostructures, including thermal,^31,32^ mechanical,^33^ optical,^32,34−37^ and phase change materials,^38^ active surrounding media under external stimulus,^22,25,39,40^ and electrical-based^41−46^ materials. The basic underlying principle of these methods relies on the high sensitivity of the nanogap plasmon resonances to small changes in the optical or geometrical properties of the nanogap region.^6^ Among these methods, electrical tunability is the most preferable approach for on-chip and information/communication technologies. Recently, electrical tuning was investigated using electrical gating configurations.^41,42^ For example, Kim et al.^42^ demonstrated an electrically controlled plasmonic response of a hybrid graphene–gold nanorod system at near-infrared wavelengths, while Qian et al.^41^ explored the electrical modulation of the plasmonic response of a hybrid graphene–silver nanowire structure at visible wavelengths. Emani et al.^46^ showed efficient electrical control of Fano resonances at near-infrared wavelengths using a multilayer graphene field effect transistor configuration. Miyata et al.^45^ successfully formed electromechanically controlled nanogaps between a gold nanowire and a gold film. Hoang et al.^44^ demonstrated electrical tuning of the plasmon response of an ensemble of nanopatch antennas embedded in an ionic liquid. Here, the tuning was achieved by swelling and deswelling the nanogap region via applying an electric potential across the antenna’s gold film and the ionic liquid. Another promising mechanism to electrically tune the optical response of a plasmonic nanogap is the Stark effect, which has greater integration potential. The Stark effect relies on modifying the transition energies of a material by applying an electric field. Consequently, this alters how the material absorbs, emits, reflects, transmits, and scatters light.^47^ The field dependence of the transition energies *E* can be expressed as^47^
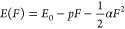
1where *F* is the electric field, *E*_0_ is energy in the absence of the electric field, *p* is the permanent dipole moment of the material, and α is the polarizability of the material.

Integration of the Stark effect with a plasmonic nanogap offers a direct method to probe the excitonic properties^47^ and morphology-correlated distribution of charge carriers and electric field^48,49^ of semiconducting materials with spatial resolution below the diffraction limit.

**Figure 1:**
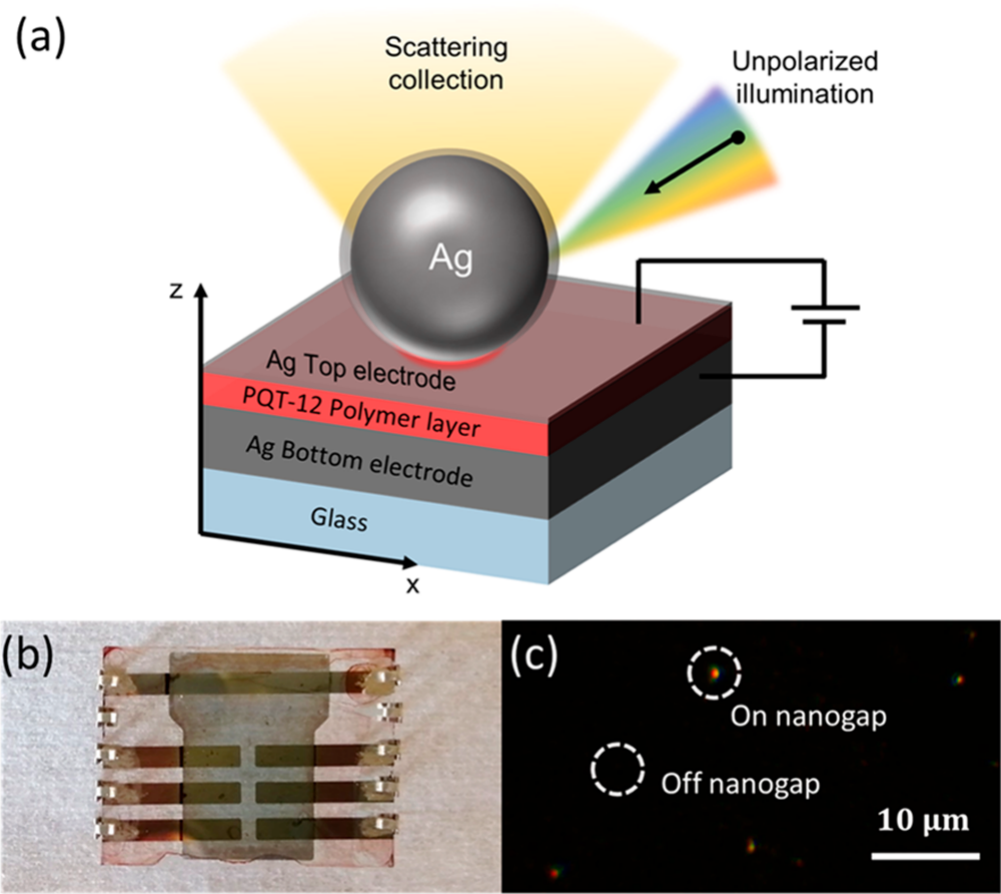
(a) Schematic representation of the electrically tuned plasmonic nanogap and the dark-field scattering setup. (b) Optical image of the fabricated devices. (c) Typical dark-field image of the device surface.

A number of different plasmonic nanogaps have been developed over the years with interesting optical properties.^6,13,50,51^ Among them, plasmonic nanogaps based on the coupling between a metallic nanostructure and a continuous metallic film have attracted much attention for their ease of incorporating electrical contacts.^44,52^ In this work, we report on using the Stark effect to control the scattering response of a plasmonic nanogap formed between a silver nanoparticle and an extended silver film separated by a 20 nm gap of the organic semiconductor (conjugated polymer) PQT-12 (poly[bis(3-dodecyl-2-thienyl)-2,2′-dithiophene-5,5′-diyl]) (see Figure 1a). The constructed plasmonic device can be utilized as an electrically tuned multiband nanoscattering source. Both observed plasmonic modes are red shifted with electric field according to a quadratic Stark shift. The approach developed in this work provides a promising way for achieving electrically tuned plasmonic devices for active nanopixels and real-time sensing applications. Furthermore, our work provides new means to interrogate the excitonic properties of organic and inorganic semiconductors and 2D materials.

## 2 Materials and Methods

### 2.1 Nanogap Fabrication

The plasmonic nanodevice consists of a glass substrate coated with a 100 nm thick layer of silver forming the device bottom electrode on top of which a 20 nm of PQT-12 was deposited by spin coating 10 g·L^–1^ of PQT-12 (Luminescence Technology Corp.) in toluene at a speed of 1500 rpm for a 50 s. To complete the plasmonic nanogap, spherical silver nanoparticles of 100 nm diameter (Sigma-Aldrich) suspended in an aqueous solution at a concentration of 2 × 10^–2^ g·L^–1^ were spin coated onto the conjugated polymer surface. Top electrical contact was achieved by thermally evaporating a 15 nm silver layer on top of the plasmonic nanogap as schematically illustrated in Figure 1a. Here, the material and geometrical parameters of the nanogap were chosen to maximize the spectral overlap between the plasmonic modes and the absorption spectrum of the PQT-12.^53^ An optical image of the fabricated electrically driven nanogap devices is shown in Figure 1b alongside a typical dark-field image of the device surface, Figure 1c.

### 2.2 Scattering Measurements

In the scattering measurements, the nanogap was illuminated by an unpolarized white light at an angle of 45° relative to the surface normal using a long working distance 10× Mitutoyo objective of numerical aperture NA = 0.28 (see Figure 1a). The scattered light from the nanogap was collected at normal incidence using a 50× Mitutoyo objective lens with NA = 0.55 (see Figure 1a). The signal was then directed toward an iHR320 Horiba spectrometer, where it was dispersed using a 150 lines/mm grating onto a liquid-nitrogen-cooled Symphony CCD. Spectra were normalized over the lamp response using a perfect scatterer.^54^ Electrical excitation was achieved using a HP 4041B picoammeter.

### 2.3 FDTD Calculations

Numerical simulations of the optical response of the investigated plasmonic devices were performed using Lumerical FDTD Solutions software. The real and imaginary parts of the polymer refractive index were retrieved from the literature.^55^ We used perfectly matching layers as boundaries. A refined uniform mesh was used over the whole total field scattered field source including the structure. In the calculations, the incident light impinged at an angle of 45° from the surface normal, matching the experimental configuration. The surface charge distribution was calculated according to the formalism described previously.^56^ TM and TE polarizations were probed separately and then averaged to present unpolarized illumination.

### 2.4 DFPT Calculations

Density functional perturbation theory (DFPT) response calculation was performed to determine the polarizability Cartesian tensor of the PQT-12 polymer. Here, periodic density functional theory (DFT) was used to describe the electronic structure of the conjugated polymer, as implemented in version 4.5 (dev 35) of the ab initio pseudopotential plane-wave package Car–Parrinello Molecular Dynamics (CPMD).^57^ We used both the PBE^58^ and the PBE0^59^ functionals with norm-conserving Goedecker (GTH) pseudo potentials^60,61^ with a plane-wave energy cutoff of 1633 eV. We account for dispersion corrections using the DFT-D2 protocol^62^ during the geometry optimizations. For all calculations, the maximum gradient component of the wave function was converged to below 10^–7^ H, and the geometry optimizations were terminated when the maximum component of the nuclear gradient was below 5 × 10^–4^ H/bohrs.

The polarizability tensor for the system was computed within the density functional perturbation theory (DFPT) framework using the implementation described in the work of Putrino et al.^63^

**Figure 2:**
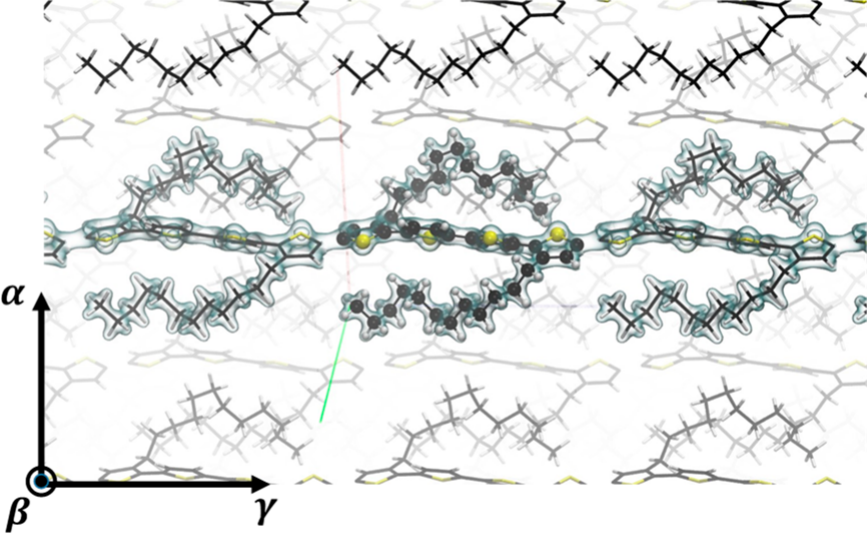
Depiction of the polymer model used for polarizability calculations, optimized at the PBE0-D2 level of theory. Unit cell contains only the atoms shown in ball-and-stick representation, while the rest of the polymer is generated using periodic boundary conditions in all directions. Axes are also shown on the figure to indicate that the γ axis correspond to the polymer axis.

The structure of the used terthiophene monomer was optimized at both the PBE-D2 and the PBE0-D2 levels of theory in a periodic unit cell that mimics an infinite chain polymer. The dimensions of the unit cell used are *A* = *B* = 16 Å and *C* = 15.3696 Å. The C–C bond that links the monomers was placed at the edge of the unit cell, along the γ (*C*) direction, while the *A* and *B* (α and β) cell vectors were set to leave enough space between chains to minimize strong interactions. The resulting optimized PBE0-D2 structure is shown in Figure 2.

## 3 Results and Discussion

**Figure 3:**
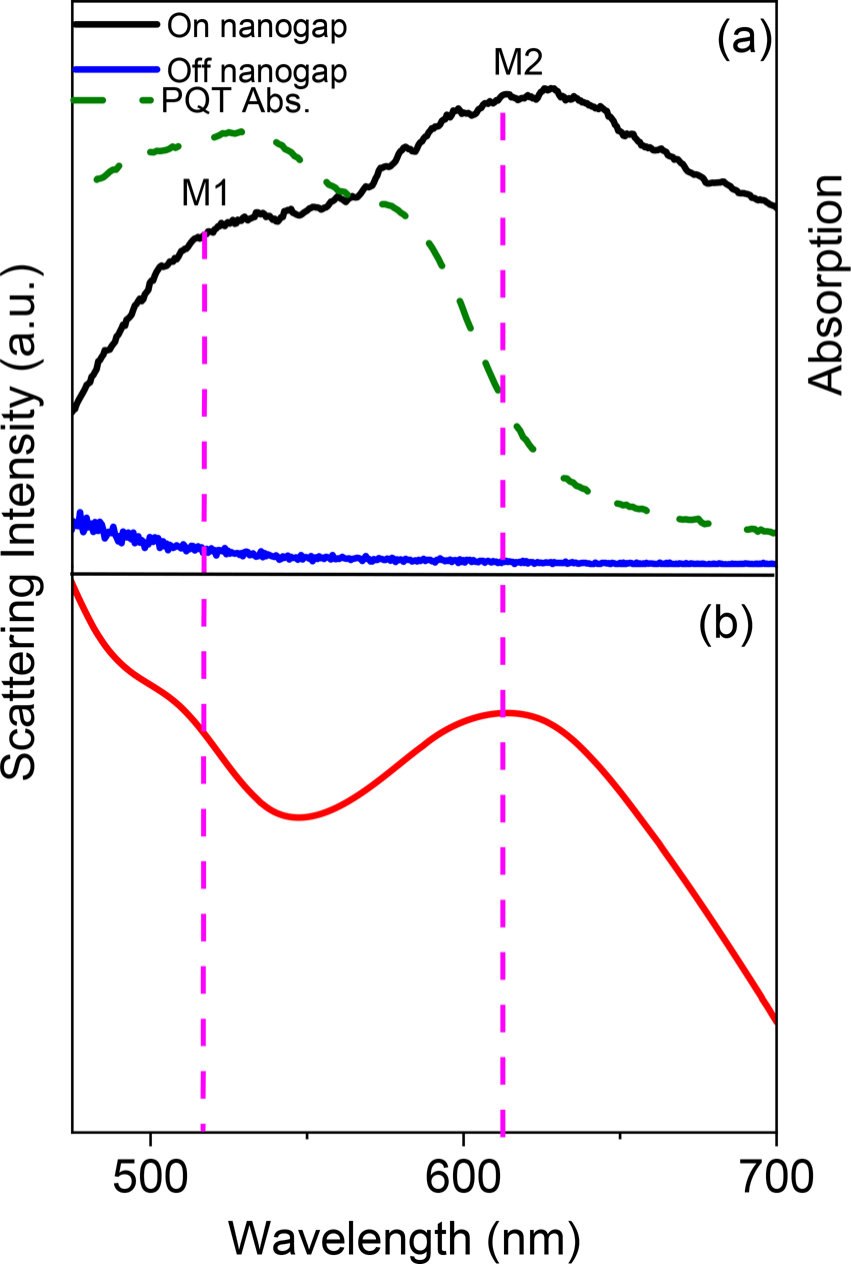
(a) Measured dark-field scattering spectra taken from the nanogap (black line) and away from the nanogap region (navy-blue line) under unpolarized illumination. PQT-12 absorption spectrum (dashed green line). (b) Calculated nanogap scattering spectrum.

Figure 3a displays two representative scattering spectra on and off the nanogap region (see also Figure 1c) at zero applied electric field, which provide us with the intrinsic scattering properties of the nanogap. On the nanogap, the spectrum exhibits two plasmon modes labeled as M1 and M2, while the spectrum off the nanogap is featureless. This clearly indicates that the measured scattered signal stems from the nanogap. Here, the plasmonic modes spectrally overlap with the absorption spectrum of PQT-12, allowing for the plasmonic response of the device to be tuned with applied electric field via changes in the absorption (transition energies) of the organic semiconductor.

To get further insight into the observed modes in Figure 3a, we calculated the optical properties of our structure using the finite difference time domain (FDTD) method. Figure 3 shows a comparison between the measured (Figure 3a) and the calculated (Figure 3b) scattering spectra for unpolarized excitation. Both spectra feature similar scattering maxima in the considered spectral region, at 510 (M1) and 620 nm (M2).

**Figure 4:**
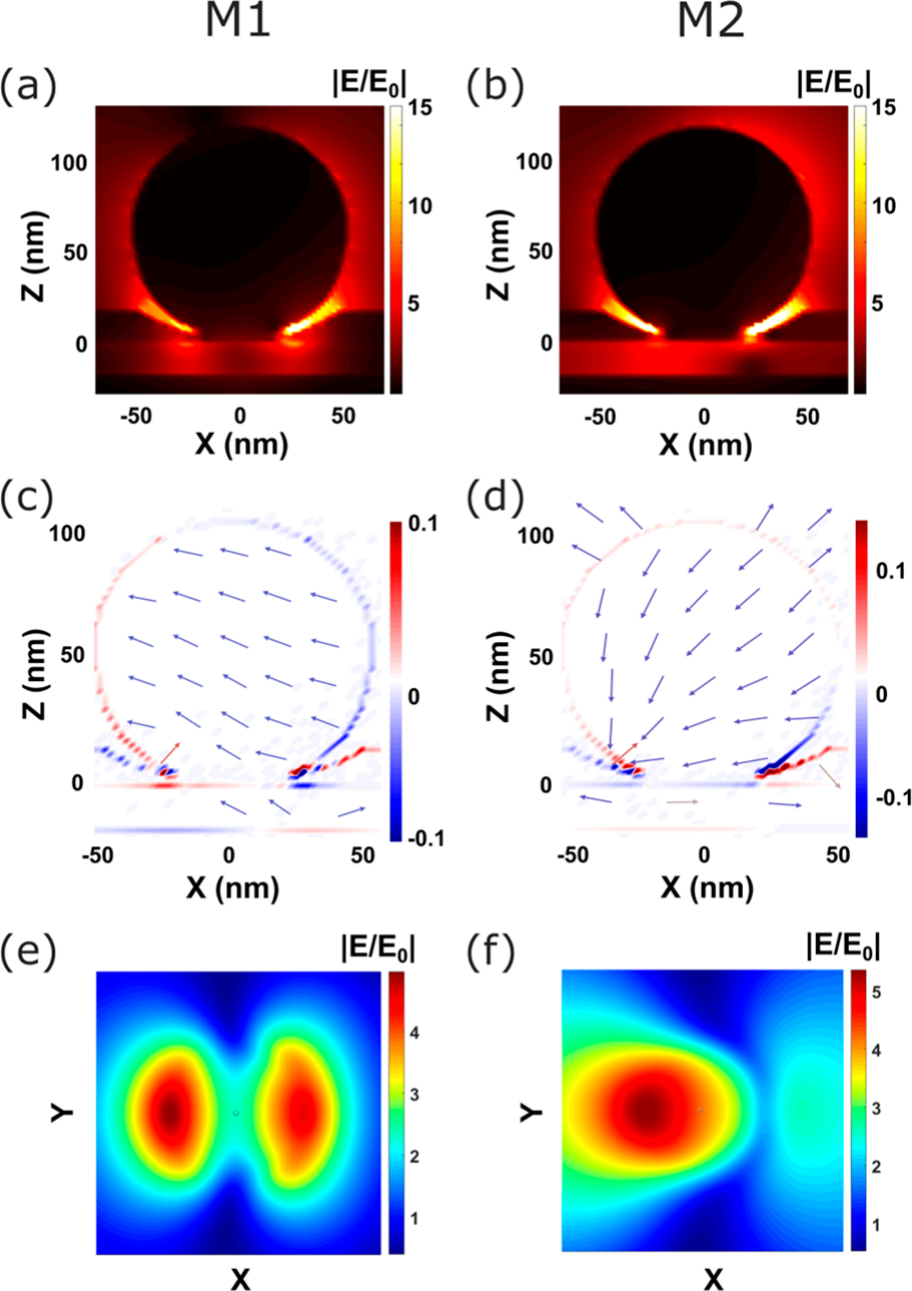
(a, b) Electric field maps: *xz* cut profiles at the center of the plasmonic nanogap for M1 (a) and M2 (b) modes under the tilted excitation. (c, d) Charge distributions and electric field vectors for M1 (c) and M2 (d). (e, f) Near-field |*E*/*E*_0_| in the middle of the nanogap for M1 (e) and M2 (f) modes.

In Figure 4a and 4b, we plot the spatial distribution of the electric field enhancement |*E*/*E*_0_| in the *x*–*z* plane of the device for wavelengths corresponding to modes M1 and M2, respectively, with both modes associated with strong field enhancement localized in the nanogap region. The nature of each mode can be determined by examining the charge distribution and electric field direction (Figure 4c and 4d), with M1 corresponding to a horizontal dipolar mode and M2 to a vertical dipolar mode. It is also important to note that the M2 mode is excited due to the vertical component of the tilted incident light in TM polarization and cannot be excited at normal incidence. However, in our structure, we also have coupling between the localized plasmonic modes and the continuum of metal–semiconductor–metal gap modes. The resulting modes in such structures are often described as cavity modes due to specific field distributions in the gap area (Figure 4e and 4f), usually labeled (*l,m*), in analogy to spherical harmonics. In these terms, M1 has the field distribution of a (1,1) mode and M2 approaches a (1,0) mode.^64^ The field distributions are slightly asymmetric due to the side illumination.

**Figure 5:**
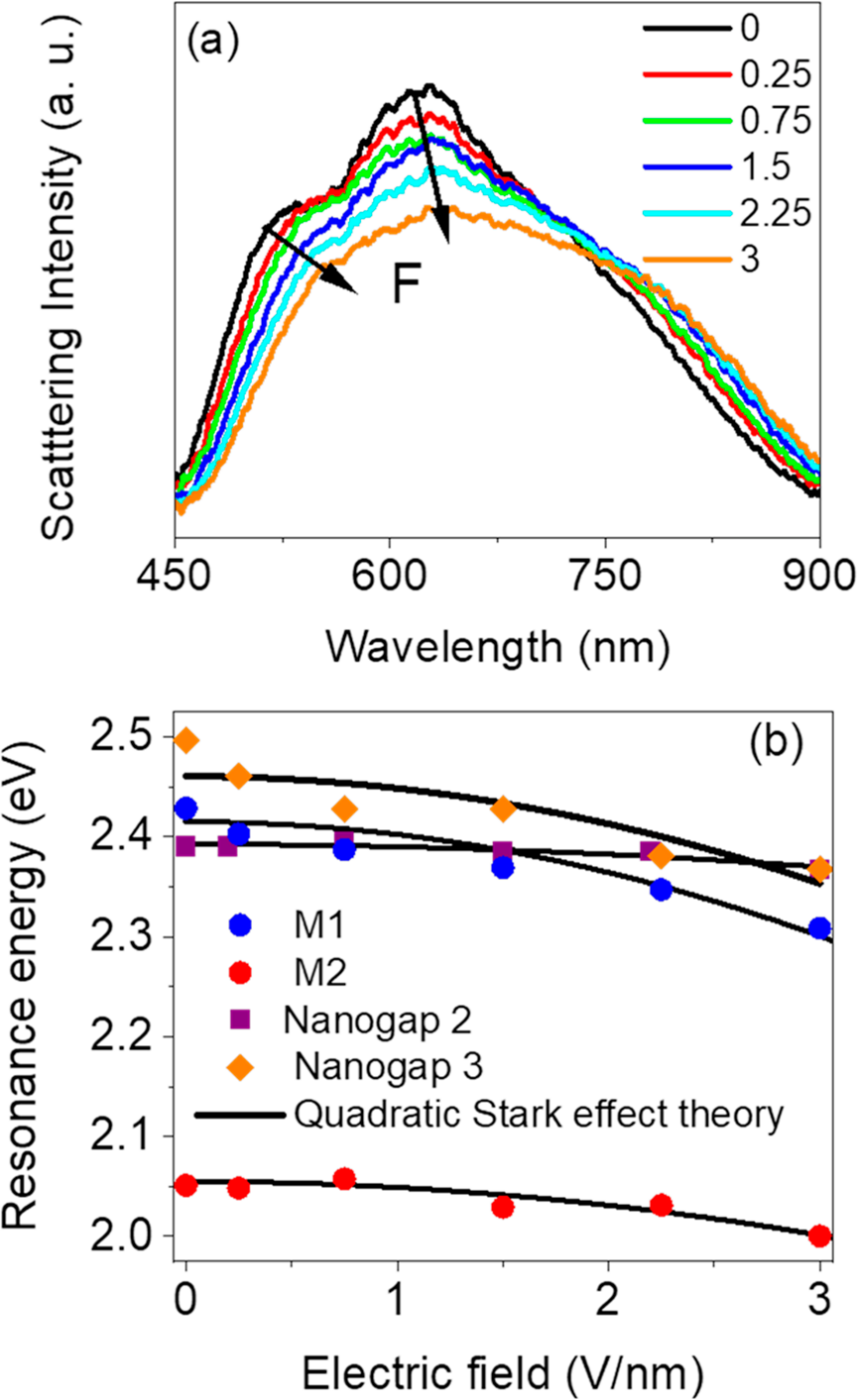
(a) Measured dark-field scattering spectra as a function of applied electric field. (b) Scattering peak position as a function of applied electric field for modes M1 and M2, alongside the scattering peak energies for two additional nanogaps. Solid curves are quadratic fits according to Stark shift dependence in conjugated polymers .

To explore the tunability of our device, we applied an external electric field across the nanogap region, with the upper 15 nm silver layer used as the top electrode and the 100 nm silver film as the bottom electrode (see Figure 1a). The externally applied electric field *F* was varied between 0 and 3 V·nm^–1^. Figure 5a displays scattering spectra of the nanogap junction for different values of the applied electric field. With increasing applied electric field, modes M1 and M2 are red shifted, with maximum shifts of 26 nm and 15 nm for M1 and M2, respectively.

To further analyze the dependence of modes M1 and M2 on the applied electric field, we plot their energy as a function of the external electric field (Figure 5b). The mode energy can be seen to shift nonlinearly with electric field, a general behavior that can be attributed to the nature of the nanogap region and how it responds to an electric field. Since the organic semiconductor PQT-12 is a conjugated polymer, it is characterized by a large oscillator strength and large polarizability, making it sensitive to externally applied electric fields, leading to a Stark effect.^65^ The resulting modification of the transition energies under applied electric field alters the material absorption of light. This modification of the absorption properties of the system can be directly coupled to a change in refractive index via the Kramers–Kronig relation.^47^ Furthermore, linear Stark shifts (arising from the term *pF* in eq 1) are not common in polymeric semiconductor ensemble measurements,^66^ and thus, the main dominant term for the Stark effect in conjugated polymers is the quadratic Stark shift
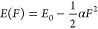
2Analysis of our experimental data shows that it closely follows the quadratic Stark shift (the solid line in Figure 5b). Furthermore, fitting the experimental data with eq 2 allows one to determine the polarizability α, yielding α_1_ = 3.7 × 10^–23^ cm^3^ for M1 and α_2_ = 1.7 × 10^–23^ cm^3^ for M2. In addition, the averaged polarizability value for the whole system was calculated to be ⟨α⟩ = 2.7 × 10^–23^ cm^3^, which is in line with the measured polarizability for other conjugated polymers such as P3HT,^67^ PCDTBT,^47^ and MEH-PPV.^68^ We also note the Stark shift associated with each plasmonic mode corresponds with their respective spectral overlap with the semiconductor absorption: with M1 shifting by 26 nm compared to 15 nm for M2.

The scattering peak energies for two additional nanogaps of similar nanoparticle size on the same sample (nanogap 2 and nanogap 3) are plotted in Figure 5b as a function of the applied electric field *F*. Their scattering energies exhibit two different values for the polymer polarizability of 0.7 × 10^–23^ cm^3^ and 3.4 × 10^–23^ cm^3^, respectively. These differences in the polarizability can be attributed to the inhomogeneity and molecular conformation of the polymer on the local level.^48,49,69,70^

We also performed a density functional perturbation theory (DFPT) response calculation to determine the polarizability Cartesian tensor of the PQT-12 polymer using both PBE and PBE0 geometries and the corresponding functional. The values obtained are shown in Table 1. We observe that both functionals give qualitatively similar results, with the PBE values being larger than the hybrid DFT values. A recent study by Hait and Head-Gordon^71^ showed that PBE typically deviates by about 10% from reference values, while PBE0 greatly improves predictions (typically only 4% deviation). Because spin-coated polymers align their molecular chains parallel to the substrate^69^ and the applied electric field in our experiment is perpendicular to the substrate, we are effectively probing the αα and ββ components of the polarizability tensor (see Figures 1a and 2). Consequently, we average the αα and ββ components of the tensor, leading to values of 7.35 × 10^–23^ cm^3^ for PBE and 5.81 × 10^–23^ cm^3^ for PBE0 for the polarizability perpendicular to the polymer propagation axis, which are in line with the measured value of PQT-12 polarizability.

**Table 1:** Polarizability Tensor Values Expressed in Units of 10^–24^ cm^3^^a^

functional	αα	αβ	αγ	ββ	βγ	γγ	⟨αα+ββ⟩
PBE	**66.1**	–5.3	1.3	**80.8**	–0.8	**205.7**	73.5
PBE0	**53.0**	–2.8	1.1	**63.1**	–0.6	**107.3**	58.1

a The tensor is symmetrical, and the diagonal values of interest are shown in bold.

The approach presented in this work is not limited to organic semiconductors but can be applied to a wide range of materials including inorganic semiconductors, quantum dots, and 2D materials, providing a new method to probe their excitonic properties at the nanoscale.

## 4 Conclusion

In conclusion, we have shown that the Stark effect can be utilized to actively control the scattering response of a plasmonic nanogap formed between a silver nanoparticle and an extended silver film separated by a thin layer of the organic semiconductor PQT-12. Under applied electric field, the scattering spectra follow a quadratic Stark shift with a maximum observed red shift of 26 nm. In addition, our approach allows for the experimental determination of the polarizability of semiconductor materials embedded in a nanogap region. Consequently, the results presented in this work not only provide a promising way for achieving electrically tuned plasmonic devices for active nanopixels and real-time sensing applications but also offer a new approach to interrogate the excitonic properties of semiconductor materials at the nanoscale.

## Abbreviations

FDTD: finite difference time domain
DFPT: density functional perturbation theory
DFT: Density functional theory
